# Hospital transfer rates and advance care planning following a nursing home-targeted video-conference education series (Project ECHO): a prospective cohort study

**DOI:** 10.1007/s41999-022-00624-6

**Published:** 2022-04-19

**Authors:** Michael J. Dowling, Una Molloy, Cathy Payne, Sarah McLean, Regina McQuillan, Claire Noonan, Dan J. Ryan

**Affiliations:** 1 Age-Related Healthcare Department, Tallaght University Hospital, Dublin 24, Ireland; 2 St. Francis Hospice, Dublin, Ireland; 3 All-Ireland Institute of Hospice and Palliative Care, Dublin, Ireland; 4 Department of Medical Gerontology, Trinity College Dublin, Dublin, Ireland

**Keywords:** Nursing home, Long-term care, Education, Advance care planning, Tele-education, Palliative care

## Abstract

**Aim:**

What effect does a novel education programme have on emergency hospital transfers of, and advance care planning decisions among, nursing home residents?

**Findings:**

This education programme did not affect overall rates of emergency hospital transfer. It did increase advance care planning discussions, increase compliance with the results of these discussions and increase “DNR” orders among nursing home residents.

**Message:**

Novel tele-education programmes have the potential to improve advance care planning discussions in nursing homes.

**Supplementary Information:**

The online version contains supplementary material available at 10.1007/s41999-022-00624-6.

## Abstract (article highlights)

**Purpose:**

Nursing home staff manage increasingly complex patients yet struggle to access education. This study measured the impact of a novel education programme on emergency transfers from nursing homes.

**Methods:**

In this prospective experimental cohort study, ten interactive sessions were provided to 20 nursing homes, using teleconferencing technology through the “Project ECHO” (Extension for Community Healthcare Outcomes) model. Details of all emergency hospital transfers were submitted by participating nursing homes 6 months before and 6 months from commencement of ECHO.

**Results:**

Of 20 nursing homes, 13 submitted sufficient data for inclusion. In these 13, there were 260 emergency transfers over a year. There was no significant difference in the number of transfers before and after ECHO (137/260 pre-ECHO vs 123/260 post-ECHO, *p* = 0.62). Post-ECHO, it was 50% more likely that transfer wishes were discussed in advance of transfer (62 of 137 (45%) transferred pre-ECHO vs 82 of 123 (67%) post-ECHO, *p* < 0.001). There was a significant increase in compliance with resident wishes post-ECHO in that transferred residents were less likely to have a documented “Not for Transfer” wish (29/137 pre-ECHO (21%) vs 10/123 post-ECHO (8%), *p* < 0.001). Point prevalence surveys of residents demonstrated significant increases in “Do Not Resuscitate” orders; 286/589 (49%) residents pre-ECHO vs 386/594 (65%) post-ECHO, *p* < 0.001. Post-ECHO, pain was less frequently the primary cause for transfer (11/137 (8%) pre-ECHO vs 1/123 (0.8%) post-ECHO, *p* = 0.006).

**Conclusion:**

ECHO did not affect rates of emergency hospital transfers but did increase advance care planning discussions ahead of hospital transfer by 50% and compliance with the results of those discussions.

**Supplementary Information:**

The online version contains supplementary material available at 10.1007/s41999-022-00624-6.

## Introduction

Nursing homes today care for patients with more comorbidities and more complex care needs than ever before. Such complexity combined with greater regulatory scrutiny culminates in increasing rates of hospital admission, often without adequate advance care planning discussion.

Nursing home residents account for a significant proportion of Emergency Department (ED) attendances; 1.9% in one American study [1]. According to another American study, nursing home residents are transferred to hospital between 3 and 16 times per person year alive [2]. This same study suggests up to half of transfers may be avoidable. Many of those transferred may have terminal illnesses with palliative needs that could be met in their nursing home. Adequate education may facilitate this; however, surveys of nursing home staff consistently highlight a dearth of education in this area [3] 4. Adequate training in advance care planning is also found lacking [5]. Qualitative data from Canadian nursing homes suggests that enhanced staff training in the palliative care topics could help avoid consequences, such as the late initiation of palliative care [6].

Consequently, there has been much study on interventions aiming to reduce emergency transfer of nursing home residents to hospital [7–9]. A common theme among these studies is the development of a new process, tool or care pathway which is then implemented within the nursing home. However, these have failed to demonstrate any significant reductions in transfers [8–10]. Studies have also struggled with implementation [9] or failed in other primary end points, such as improving residents’ comfort in the last week of life [11]. INTERACT is a programme that provides tools and strategies to assist nursing home staff in early identification, communication, and documentation of changes in resident status [10]. Although a pilot study led to a reduction in all cause hospitalisations from nursing homes [12], subsequent implementations of this programme in a randomised study [8] and in other jurisdictions [10] did not show significant ED attendance reductions.

Although there is a paucity of research on whether palliative care education can reduce hospital transfers and enhance advance care planning, qualitative research in Sweden reported that registered nurses felt supported in their decisions not to transfer older persons to hospital if the nursing home has a palliative approach, or if documented advance care plans exist [13]. SARS-COV-2-related pandemic waves have necessitated advance care planning together with complex palliative care and medical management in nursing homes in an effort to maintain holistic care and also react to the ethics of distributive justice in a setting of limited hospital resources. Our education programme seeks to teach nursing home staff on topics related to palliative care to better equip them to care for their residents and implement processes that are potentially more locally applicable. This education based approach; although described [14], is much less common in the literature than studies on the implementation of new processes, tools or pathways etc. within nursing homes.

Social distancing measures in a pandemic era are a further obstacle to face-to-face education and have expedited audio–visual telecommunication education programmes globally. They have utilised varying education methods with varying degrees of engagement and success yet to date there is limited outcome data on learning objectives, advance care planning and transfer decisions. Project ECHO (Extension for Community Healthcare Outcomes) is an educational method that has gained international traction. Experts at “hub” sites use teleconferencing technology to facilitate educational sessions with caregivers at “spoke” sites, with the emphasis on peer learning and “spoke–spoke” interaction. Project ECHO has been used in 39 countries, and has facilitated teaching in 77 topics, including infectious disease, mental health and cancer screening [15]. Two programmes were launched in Dublin, Ireland in 2016 and 2018. The first demonstrated significant improvements in staff confidence in dealing with complex palliative patients [16]. The second, this study, aimed to assess the second ECHO-delivered education programme’s effect on emergency transfers from nursing homes, advance care planning, primary reason for transfer, and length of hospital stay. We hypothesised that an education programme targeting palliative care would reduce rates of hospital transfers and would increase rates of advance care planning among nursing home residents.

## Methods

In this prospective experimental cohort study, we assessed the impact of a ten-lecture education series on nursing homes. We selected as a primary outcome hospital transfer of residents from nursing homes, on the basis that a dedicated palliative curriculum may influence transfers by affecting advance care planning and more appropriate symptom control. The secondary outcomes were defined as resident admission to hospital, length of stay in hospital, whether regular GP was involved in decision to transfer, whether out-of-hours doctor was involved in decision to transfer, weekend transfers, primary reason for transfer, and advance care planning (defined as DNR orders in place and documented resident wishes with regards to emergency hospital transfer).

In this experimental cohort study, the intervention was defined as a nursing home institution attending at least 40% of the ten-lecture series.

Criteria for inclusion was that nursing homes were located within the county of Dublin (capital city in which both “Hubs” were based) and that they mainly cared for older persons. Nursing homes could be publicly or privately funded. The 20 nursing homes that took part in an earlier ECHO-delivered palliative care education programme in 2016 were excluded to reduce bias. Thus, 79 nursing homes in the greater Dublin area were invited to participate in the education programme. All nursing homes that met the above criteria were invited, 22 were recruited from that invite.

Regarding statistical power calculation for the primary outcome, there is a dearth of literature on nursing home hospital transfer rates; however, evidence from American nursing homes reported a 15.5% hospital transfer rate within a 6-month period [17]. Although other studies have reported higher admission rates (up to 28.8% [18] annually and 37% over a 3-year period [19]), the authors used the more conservative measure of 15.5% for power calculation to maximise the chance of statistically significant results. We anticipated the recruitment of 20–30 nursing homes from the 79 invited. The 22 nursing homes that agreed to participate in the study had a combined total of 1889 residents. At the 15.5% hospital transfer rate we anticipated 293 hospital admissions from nursing homes during each 6-month period. As no standard deviation estimates were available for nursing home admission rates, we assumed a wide variability and used a standard deviation of 50% of the mean (*n* = 293). Using these assumptions, recruitment of 22 nursing homes yielded a greater than 80% power to detect a 30% reduction in hospital transfers from nursing homes during the 6-month period post-intervention. Thus, 6 months was chosen as the data collection time period pre- and post-ECHO.

The “Hubs” were St. Francis Hospice, Dublin 15 and Our Lady’s Hospice & Care Services, Dublin 6 W. Ethical approval was provided by the St. Francis Hospice Ethics Committee. An initial workshop took place, where representatives from each of the nursing homes met with experts from the hubs, and key palliative care topics were identified. Ten educational sessions then took place over the spring/summer of 2018, each focusing on one of the following topics and delivered by an expert of experts in the field (in brackets):

1. Recognising dying and prognostication (Clinical Nurse Specialists in Palliative Care).
2. Pain Assessment/Management (Palliative Care Consultant).
3. Advance Care Planning (Palliative Care Consultant).
4. Nutrition and Hydration (Clinical Nutritionist).
5. Referral to Specialist Palliative Care (Palliative Care Specialist Registrar).
6. Managing Conflict (Hospice Social Worker).
7. Team Distress (Clinical Psychologist).
8. Sedation and Delirium (Consultant Geriatrician).
9. Managing Respiratory Symptoms: Shortness of Breath and Secretions (Clinical Nurse Specialist in Palliative Care and Hospice Physiotherapist).
10. Medication Management: Anticipatory Prescribing/Medication Rationalisation (Hospice Pharmacists).

At each 90-min session, a short presentation on the topic at hand was delivered. Then, two nursing homes per session outlined a relevant case presentation. Finally, a discussion around the topic and cases was facilitated, with emphasis on peer learning and “spoke–spoke” interaction, with expert oversight. A “Q&A” on the previous session was also included.

Sessions took place using “Zoom” teleconferencing technology, which permits visualisation of all participants on the screen to facilitate spoke–spoke discussion.

**Fig. 1:**
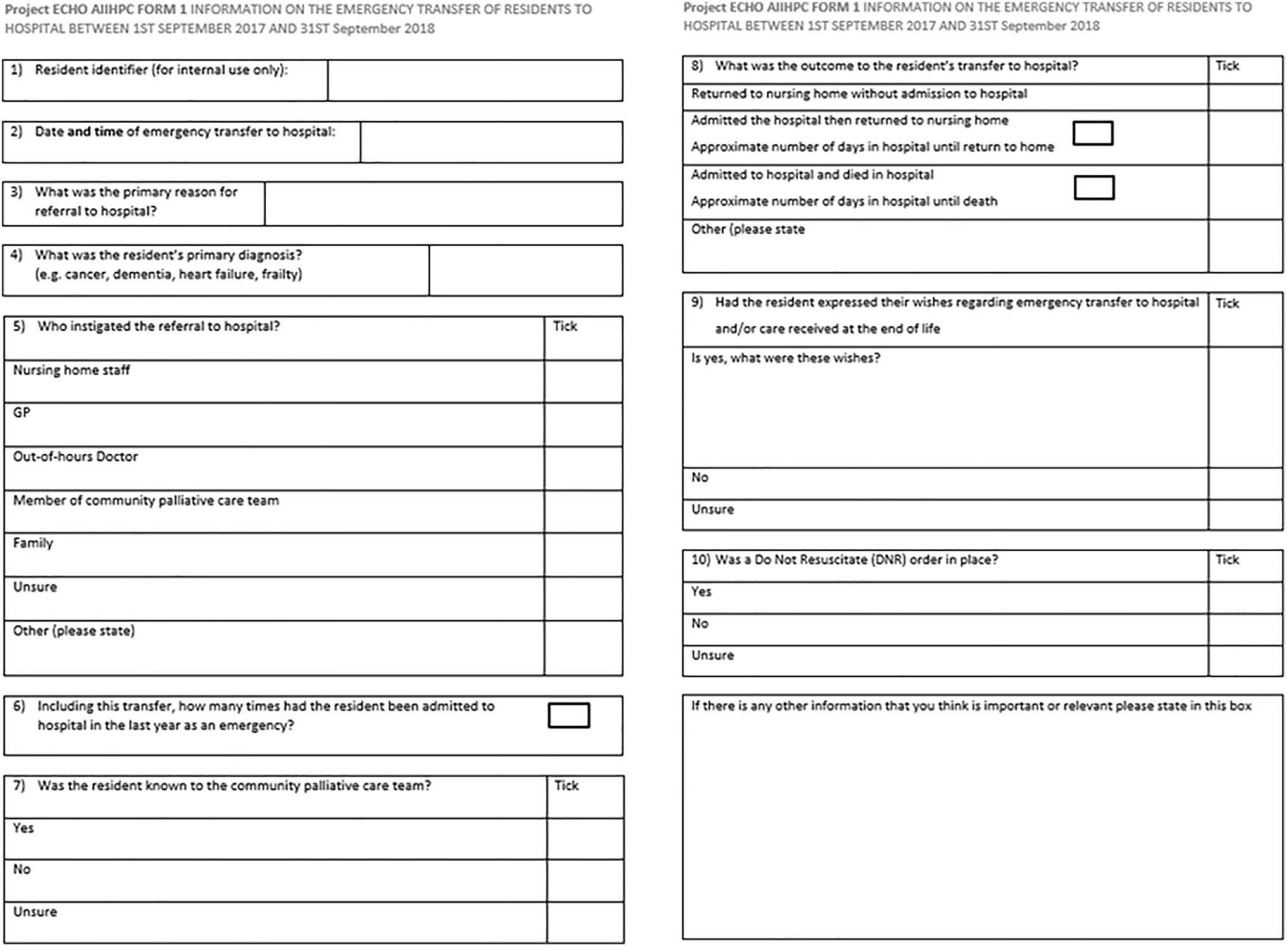
Transfer form to be completed by nursing home staff for all residents requiring emergency transfer to hospital during study period

For 6 months prior to the education programme (September 2017–February 2018), participating nursing homes were required to fill out data forms on each patient transferred to a hospital emergency department from their facility. This data collection was then repeated for the 6-month post-commencement of programme (April 2018–September 2018). The data included in this “Transfer Form” included the patient’s primary diagnosis, the reason for emergency transfer to hospital, the outcomes of the transfer and details regarding advance care planning, such as previously documented patient wishes. The entire contents of the “Transfer Form” are displayed in Fig. 1.

In addition, each nursing home was required to fill in a “Survey Form”, which included the documented wishes of all their residents (not just those transferred to hospital) at a date 6 weeks prior to the education sessions, and again at least 6 weeks after the education sessions were completed. A copy of this “Survey Form” is available in the supplementary data.

The above transfer forms and survey forms were collected by a nominated contact person in each nursing home who was the main point of contact between the nursing homes and the research team. This was, in all cases, the persons in charge at the nursing home (director of nursing or clinical nurse manager). No identifying patient details were on these data sets. To minimise the risk of bias, nursing home staff were assured that all data identifying nursing homes would be anonymised as soon as it was entered into datasheets by the research team. Data forms were securely emailed or posted to the research team. All nursing home nominated contact persons were contacted by the research team via phone call to ensure all data was collected prior to analysis.

Univariate statistical analysis of continuous parametric data was performed using Student’s *t* test, and Mann–Whitney U test for non-parametric data. Categorical data was analysed using chi-square test and Fisher’s exact test. Descriptive statistics were calculated for all extracted data and summarized in the text below and in Tables 1, 2, and 3. The data was entered into a Microsoft Excel Workbook and analysed in Stata 13.

The primary reason for transfer to hospital for each patient was analysed and grouped into categories (pain, abdominal complaint, weakness/general deterioration, confusion, respiratory complaint, fall/trauma). There is no established way of categorising hospital transfers by presentation in the literature, so the "Reason for Transfer" for each patient was assigned to the above categories by blinded consensus methodology. Two authors, each blinded to whether the patient was from pre- or post-intervention, assigned each patient to a presentation category. Disagreements were dealt with through consensus discussion.

## Results

Of the original 22 nursing homes who agreed to participate in education programme, 20 attended. Of the two that dropped out, one reported that no staff were available to attend the sessions, the other did not give a reason. Of these 20, five attended less than four of the ten educational sessions, and were excluded from analysis. Of the non-attended sessions among these five nursing homes, staff reported that no one was available 50% of the time; no reason was given for the other half. These five nursing homes did not significantly differ in size when compared to the 15 that did attend sufficient sessions (*p* = 0.87). Of the remaining 15 nursing homes, 13 submitted the requisite data described above for both pre- and post-ECHO. Again, there was no significant difference in the size of the nursing homes that did/did not submit the requisite data (*p* = 0.87). Of the seven nursing homes excluded due to poor attendance/lack of data, none were publicly funded. Of the 13 nursing homes included in data analysis two were publicly funded, while the remaining homes were privately funded. This does not deviate significantly from the nationwide proportion of publicly funded nursing home beds (18%, *p* = 0.82) [20]. These 13 nursing homes varied in size from 28 to 134 residents (IQR 77). Of the 13 nursing homes included in the analysis, each nursing home attended an average of 7.7 (SD = 2) sessions. In total, the 13 nursing homes submitted data for 260 emergency department transfers including both the pre-ECHO period and the post-ECHO period. The details of these transfers pre- and post-ECHO are summarised in Tables 1 and 2.

**Table 1:** Details of emergency nursing home patient transfers to hospital pre- and post-intervention

	Nursing homes Pre-ECHO	Nursing homes during-ECHO	*p* value
	*n* = 137	*n* = 123	
Total no. transfers/nursing home, mean (SD)	10.5 (7.5)	9.5 (6)	0.62
No. Residents transferred at least once per nursing home, mean (SD)	8.6 (6.4)	7.3 (5.2)	0.64
No. transfers admitted from emergency department, mean (SD)	8.9 (6.4)	7.3 (4.5)	0.44
Length of hospital stay for admitted patients, median (IQR)	7.2 (4)	7.8 (5)	0.75
GP involved in transfer, *n* (%)	59 (43%)	55 (45%)	0.82
Out-of-hours doctor involved in transfer, *n* (%)	18 (13%)	10 (8%)	0.28
Weekend transfers, *n* (%)	39 (28%)	33 (27%)	0.77
Pain documented as primary reason for transfer, *n* (%)	11 (8%)	1 (0.8%)	0.006

**Table 2:** Previously documented patient wishes in advance of emergency transfer

	Nursing homes Pre-ECHO	Nursing homes during-ECHO	*p* value
	*n* = 137	*n* = 123	
DNR order in place	59 (43%)	65 (53%)	0.12
Documented wish regarding emergency transfer	62 (45%)	82 (67%)	< 0.001
**Decision of those with documented wishes regarding emergency transfer**			
	*n* = 62	*n* = 82	
For transfer (% of those with documented wish)	27 (44%)	67 (82%)	< 0.001
Not for transfer (% of those with documented wish)	29 (47%)	10 (12%)	< 0.001
Resident documented wish not clear	6 (10%)	5 (6%)	0.42

There was no significant difference in the number of patients transferred to hospital pre-ECHO vs post-ECHO. Nor was there a significant difference in the number of patients admitted to hospital, nor the length of stay among those admitted pre- vs post-ECHO. There was also no significant difference in the number of weekend transfers pre- vs post-ECHO.

After the education programme it was significantly more likely that transfer wishes were discussed with the patient in advance of any impending emergency hospital transfer. Pre-ECHO, 62 of 137 (45%) transfers had their wishes discussed before transfer, while post-ECHO 82 of 123 transfers (67%) had their transfer wishes discussed in advance of transfer (*p* < 0.001). Most of the increase in advance care planning conversations resulted in a “For Transfer” decision. The number of documented “For Transfer” wishes increased from 27 of 62 (44%) pre-ECHO to 67 of 82 (82%) post-ECHO, *p* < 0.001. Correspondingly the number of documented “Not for Transfer” wishes among those transferred to hospital reduced from 29 of 62 (47%) pre-ECHO to 10 of 82 (67%) post-ECHO, *p* < 0.001.

The impact of the education programme on variation in primary reason for transfer to hospital was also analysed. There was only a significant decrease in those transferred with pain documented as the primary reason (11 of 137 (8%) pre-ECHO vs 1 of 123 (0.8%) post-ECHO, *p* = 0.006). All other categories remained constant (abdominal complaint, weakness/general deterioration, confusion, respiratory complaint, fall/trauma).

Participating nursing homes also completed a single assessment of advance care planning among all nursing home residents on a given day. The results of these forms are summarised in Table 3. After the ECHO education programme, significantly more residents had a “Do Not Resuscitate” order.

**Table 3:** Survey of all residents in participating nursing homes pre and post-intervention

	Pre-ECHO	Post-ECHO	*p* values
Total number of residents	589	594	
Number of current residents with expressed wishes regarding EOL	449 (76%)	395 (66%)	< 0.001
Number of current residents who have a DNR order in place	286 (49%)	386 (65%)	< 0.001
Number of referrals to Community Palliative Care Services in preceding 6 months	13	10	0.51

## Discussion

This was the second education programme of its kind in the Republic of Ireland, teaching palliative care skills to nursing home staff through an ECHO-delivered teleconference model. The first programme was observed to significantly increase staff confidence in managing common palliative care scenarios [16].

This prospective experimental cohort study did not influence the rates of emergency transfers from nursing homes to hospital. It did not influence rates of weekend transfers or length of hospital stay among those admitted to hospital. The lack of effect is likely attributable to factors unrelated to palliative care education and the limited impact on clinical outcomes of a ten-session programme. It has also been described in the literature that education delivered to nursing homes can effect change on an individual level that does not translate to changes on an organisation level [21], which may have been a factor in ECHO. Similar studies that sought to reduce the number of nursing home residents being transferred to the emergency department also did not succeed in their primary outcome [7–9]. It is possible that the outcomes of such interventions/education programmes are best measured in other patient/staff/institution markers, such as advance care planning or arbitrary assessment of learning outcomes. Future study in this area may benefit in targeting these markers as their primary outcome. The programme may have altered the type of presentation to hospital, in that patients post-ECHO were significantly less likely to be transferred for management of pain. This may have been because staff members felt empowered to better recognise and treat pain after our lecture series (a central principle of palliative care [22]) or it may have been a type 1 statistical error.

This data does show that a ten-session ECHO teleconference delivered education programme increased the likelihood of nursing home staff conforming to patient wishes regarding emergency transfer to hospital. Pre-intervention, 21% of patients transferred to hospital had a documented “Not for transfer” wish. This significantly dropped to 8% post-intervention. It is possible that the education programme has empowered staff to follow documented patient wishes and manage more patients whose wish it is to stay in the nursing home. ECHO also significantly increased likelihood that a “For transfer” wish was expressed among those transferred, further evidence of increasing conformity with patient wishes. However, increased discussions mostly resulted in a “For transfer” decision. Data was not collected on the nature of and attendees at advance care planning discussions. Nor is it available for “Do Not Resuscitate” discussions; however, the education programme also appears to have significantly increased the resuscitation discussions in the nursing home or at least altered the course of the discussions toward a “Do Not Resuscitate” order. Table 3 shows that post-ECHO, there was a lower number of residents with expressed wishes regarding end-of-life (EOL) care. This is difficult to explain, but it may be that the question was misunderstood by some nursing homes, and that residents who were now recorded as having a DNR order were not recorded as having expressed wishes regarding EOL care. There is evidence for this in that it is unlikely that among 395 residents with expressed wishes regarding EOL, 386 of these expressed wishes included a DNR order.

The study has several limitations. The patient transfer data collection took place during different times of the year (pre-ECHO September 2017–February 2018, post-ECHO April 2018–September 2018), with previous studies showing that a higher proportion of nursing home patients use acute hospital beds during winter months [23]. This may have skewed results when comparing total numbers of patient transfers during the two 6-month periods. The data was collected by senior nurse managers within the nursing homes, and although anonymisation was assured, as the data was not collected by a third-party observer bias is possible. The study was not resourced for independent researchers to go to the nursing homes to collect the data and thus relied on nursing managers to do this. However, the researchers had a close working relationship with nursing home managers and a member of the research team spoke directly with them in all cases in relation to data acquisition. The organisers of the lecture series provided data collection information to the nursing managers pre- and post-lecture and data collection was also discussed at the workshop stages. In addition, staff awareness of inadequate documentation after the lecture series may have given rise to a greater likelihood to describe their high rates of advance care planning after the lecture series. The research team do not have a way of arbitrarily confirming the validity of nursing home staff reporting with regards to this.

Previous studies [8] that have used hospital transfers of nursing home residents as a primary outcome have measured these outcomes by assessing Medicare (the national health insurance programme in the U.S.A.) data. It could be argued that Medicare or equivalent data is more likely to be accurate than reported patient transfers from internal staff members. However, it is not unusual in the literature for nursing home staff to participate in data collection for studies based within their own workplace [10–12]. However, some of these studies [10, 12] included data on nursing home daily censuses to confirm hospital transfers which, although self-reported, may have added to the robustness of the data.

This study was unable to assess the medium to long-term effects of this education programme, as data collection was completed less than 2 months after the programme finished. While a previous ECHO-delivered education programme showed persistent improvements in staff confidence 6-week post-intervention [16], further research is needed to determine if such a programme can have long-term effects on tangible patient outcomes. In addition, internationally, ECHO education programmes often run on a regular basis, lasting for months or years [24]. It is possible that a more long-term education programme delivered to nursing home staff could result in more meaningful effects in relation to advance care planning transfer decisions and possibly even on clinical outcomes. A literature review on nursing home staff training showed interventions with ongoing support were more likely to successfully demonstrate sustained implementation of new knowledge [25]. A further potential confounding factor is that prior palliative care education delivered to nursing homes was not formally surveyed as part of this study. Nursing homes that were included in the first phase of ECHO-delivered palliative care education in 2016 were not invited to participate in this second phase of ECHO. It is possible that other palliative care education programmes took place in sites included in this study; however, focus group discussions with directors of nursing pre-education did not reveal any. Furthermore, teleconference education was rare prior to the SARS-CoV-2-related pandemic, and it is unlikely a large-scale on-site education programme took place locally without the research consortium being aware of its presence.

Data collection was incomplete, as 5 out of 20 nursing homes did not attend sufficient sessions for data analysis, and 2 out of 15 did not submit transfer data, thus selection bias is possible. In addition, the 20 nursing homes that took part in the education series self-selected for the study from the 79 invited, a further chance of selection bias. The international literature does show that nursing homes with higher engagement in quality improvement projects to reduce hospitalisations are more effective than those with lesser engagement, and it is feasible that this finding would be replicated in education interventions, such as ECHO. A 2012 trial of the INTERACT programme to reduce hospitalisations among nursing home residents showed the highest reductions were seen in the facilities with the highest engagement with the programme [10]. The nursing homes that participated more wholeheartedly with the ECHO programme may have had intrinsic factors that meant they were more likely to see improvements among the primary and secondary outcomes.

Attendance has been cited in the international literature as a difficulty in delivering education to nursing home staff; an Australian study delivered to staff failed to improve resident quality of life; however sub-group analysis found that quality of life of residents was improved when only facilities with a greater than 50% attendance were included [26]. As well as the effects of nursing home education programmes, factors that affect and can potentially improve staff attendance at such programmes is an area, where further research is needed.

The current pandemic has demonstrated the necessity of urgent upskilling of nursing home staff in palliative medicine but also medical management and infection control. This education approach is globally validated and acknowledged in other medical fields and provides a framework for addressing the current, urgent need for palliative and medical education in long-stay facilities. It is particularly appropriate for facilities geographically remote from hospital expertise and now unable to access face-to-face education programmes. Even prior to the SARS-CoV-2-related pandemic, surveys have shown geography and time constraints to be a barrier to accessing education among nursing home staff [27]. This study describes a programme that could be relatively easily implemented in most jurisdictions, and although the study did not succeed in its primary outcome, it did improve advance care planning amongst residents, which has become all the more relevant in recent times.

## Conclusion

This ECHO-mediated, palliative care education programme did not affect rates of emergency transfer of nursing home residents, nor did it largely affect the reason for transfer. It did increase the likelihood of advance discussion regarding patients transfer wishes and adherence with that wish, and also increased the likelihood of a DNR being established within the nursing homes. Further research is needed to assess whether longer term education programmes can have sustained effects on staff competency, advanced care planning and patient outcomes.

## Supplementary Information

Below is the link to the electronic supplementary material.

Supplementary file1 (DOCX 18 KB)

## Data Availability

Available on request.
