# Supplementary material for: Hospital transfer rates and advance care planning following a nursing home-targeted video-conference education series (Project ECHO): a prospective cohort study
Source: Eur Geriatr Med. 2022 Apr 19;13(4):941–9. doi: 10.1007/s41999-022-00624-6 (PMC9016377; doi:10.1007/s41999-022-00624-6)
Supplement: Supplementary file 1 — Supplementary file1 (DOCX 18 KB) [file 41999_2022_624_MOESM1_ESM.docx]

**Article Title -** A nursing home-targeted video-conference education series (Project ECHO) improved advanced care planning among those transferred to hospital but did not reduce rates of hospital transfer.

**Journal Name** – European Geriatric Medicine

**Author Information**

Dowling Michael J^a^ (MB MRCPI) Orcid 0000-0002-5227-1852

Molloy Una^b^ (RGN PhD)

Payne Cathy^c^ (PhD RD)

McLean Sarah^b^ (MB MRCPI MSc)

McQuillan Regina^b^ (MB MRCPI)

Noonan Claire^a^ (RANP RGN Msc HDip)

Ryan Dan J^ad^ (MB MRCPI PhD)

1. Age-related Healthcare Department, Tallaght University Hospital, Dublin, Ireland
2. St. Francis Hospice, Dublin, Ireland
3. All-Ireland Institute of Hospice and Palliative Care, Dublin, Ireland
4. Department of Medical Gerontology, Trinity College Dublin, Dublin, Ireland

**Corresponding author**

Michael Dowling, Age-related Healthcare Dept, Tallaght University Hospital, Dublin 24, Ireland. dowlinmi@tcd.ie. 00353861556062

**Supplementary Data – Point prevalence “Survey Form” capturing wishes of residents**

Project ECHO AIIHPC FORM 2 Information on current residents residing in the nursing home. To be completed for a single day between 20th August and 24th August.2018

| 1. Date of completion |  |
| --- | --- |

| 1. Total number of residents |  |
| --- | --- |

| 1. Total number of current residents who have a documented expression of their wishes regarding care at the end of life, including their preferences for medical interventions |  |
| --- | --- |

| 1. Total number of current residents who have a Do Not Resuscitate (DNR) order in place |  |
| --- | --- |

| 1. Total number of referrals to community palliative care services since 1st April 2018 |  |
| --- | --- |

| 1. Total number of resident deaths since 1st April 2018 |  |
| --- | --- |

| If there is any other information that you think is important or relevant, please state in this box |
| --- |
